# Case Report: Clinical Remission in a Cat With Severe Bilateral Eosinophilic Keratitis Receiving Combined Immunosuppressive Therapy (Triamcinolone Acetonide and Tacrolimus)

**DOI:** 10.3389/fvets.2021.580396

**Published:** 2021-04-30

**Authors:** Amanda K. Romaneck, Lionel Sebbag

**Affiliations:** ^1^Department of Veterinary Clinical Sciences, College of Veterinary Medicine, Iowa State University, Ames, IA, United States; ^2^Koret School of Veterinary Medicine, The Hebrew University of Jerusalem, Rehovot, Israel

**Keywords:** eosinophilic keratitis, eosinophilic keratoconjunctivitis, triamcinolone acetonide, corticosteroids, immunomodulation, antiviral drugs, compliance

## Abstract

A 2-year-old domestic shorthair cat was examined for severe keratitis of 10 months duration, non-responsive to 0.1% dexamethasone q8-12h. Patient and owner compliance were poor given ocular discomfort and hiding behavior. On presentation, both eyes (OU) had severe ulcerative keratitis that masked examination of deeper structures and resulted in absent menace response OU. Corneal cytology was consistent with eosinophilic keratitis (EK) OU. Initial therapy included subcutaneous triamcinolone acetonide injection (0.2 mg/kg), 0.3% ciprofloxacin OU q12h, and two compounded drugs started 5 days later upon receipt: 0.5% tacrolimus OU q6h, 0.5% cidofovir OU q12h. Visual behavior and ocular comfort were reportedly much improved within 24 h. At the first recheck (Day 11), patient and owner compliance were reported to be excellent, menace response was positive OU, and keratitis was dramatically reduced OU with absent fluorescein uptake. Subcutaneous triamcinolone acetonide was repeated (0.2 mg/kg), ciprofloxacin was discontinued, cidofovir was continued q12h, and tacrolimus was slowly tapered (q8h × 3 weeks then q12h). Keratitis was nearly 100% resolved at the second recheck (Day 38); cidofovir was discontinued and tacrolimus was slowly tapered (q12h × 1 week, q24h × 4 weeks, q48h × 4 weeks) then discontinued. A third recheck (Day 101) confirmed clinical remission OU, and disease did not reoccur within a 1 year follow up period (photographic documentation by owner). In sum, adjunct use of triamcinolone acetonide greatly improved therapeutic compliance and helped control severe EK in a rapid and effective manner.

## Background

Eosinophilic keratitis (EK) is a chronic and progressive keratopathy described in cats and other species, characterized by proliferative white to pink granular corneal plaques and corneal neovascularization (1–5). The exact pathogenesis of EK is unknown, although an association with feline herpesvirus type 1 (FHV-1) has been proposed in cats (6), and the cellular profile is typically consistent with either a type I (IgE-mediated) or type IV (T-lymphocyte-mediated) hypersensitivity reaction (7).

Diagnosis of EK can be confirmed with corneal cytology—revealing a predominantly eosinophilic inflammation—while treatment of EK is aimed at controlling the immune-mediated disease with the use of topical and/or systemic immune-modulatory drugs. Several challenges complicate the clinical management of EK in cats, including the balance between local immunosuppression and FHV-1 reactivation (8), risk of topical corticosteroids in the presence of corneal ulceration (reported in 66.7% of EK cats) (3), poor therapeutic compliance long-term (2), and relatively high rate of recurrences (up to 66%) (1).

The purpose of the present case report is to describe the successful management of severe bilateral EK in a cat, detailing a unique protocol that enhanced therapeutic compliance, improved clinical signs in a rapid manner, and provided long-term resolution without maintenance immunomodulation (clinical remission at 1 year follow up).

## Case Presentation

Written informed consent was obtained from the owner for the publication of this case report. A 2-year-old male castrated domestic shorthair (7 kg) presented for evaluation of keratitis of approximately 10 months duration. The cat was otherwise healthy with no known systemic diseases, and he was up-to-date on vaccines and flea and tick prevention. An opacity was first noted by the owner in the lateral aspect of the cornea in the left eye (OS), followed weeks later by similar changes in the right eye (OD). The lesions slowly extended over the entire corneal surface in both eyes (OU) over several months, resulting in visual impairment, blepharospasm, ocular discharge, and redness. According to the owner, the visual impairment affected the cat's general demeanor (i.e., hiding behavior, easily startled) and started disrupting the human-animal bond in the household. Two months prior to presentation, the cat was prescribed topical 0.1% dexamethasone (Maxidex, Novartis, Basel, Switzerland) by the referring veterinarian with a recommended administration frequency of three times daily OU. However, therapy was only given twice daily by the owner due to the patient's ocular discomfort and poor compliance, and no improvement was noted at the completion of the 3-week course.

**Figure 1:**
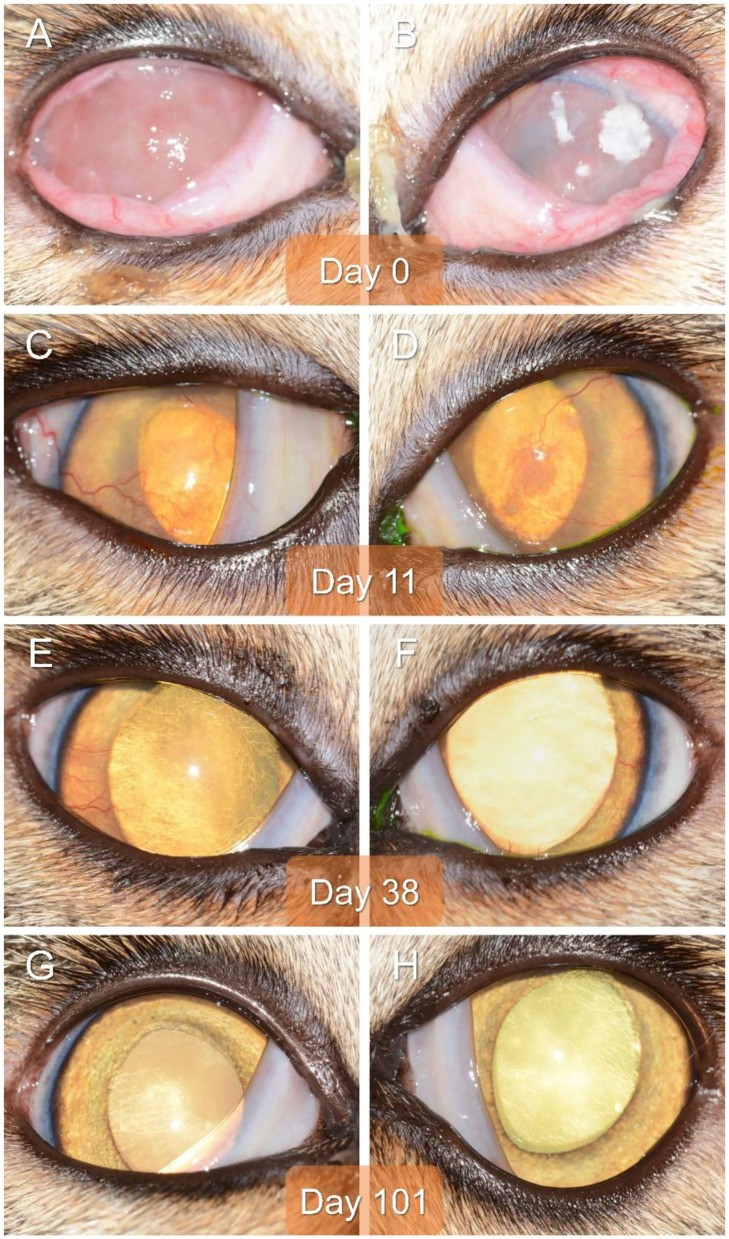
Clinical images of the right eye **(A,C,E,G)** and left eye **(B,D,F,H)** of a 2-year-old domestic shorthair cat diagnosed with severe bilateral eosinophilic keratitis, managed with combined immunosuppressive therapy (subcutaneous triamcinolone acetonide and topical 0.5% tacrolimus), and examined on Day 0 **(A,B)**, Day 11 **(C,D)**, Day 38 **(E,F)**, and Day 101 **(G,H)**.

**Figure 2:**
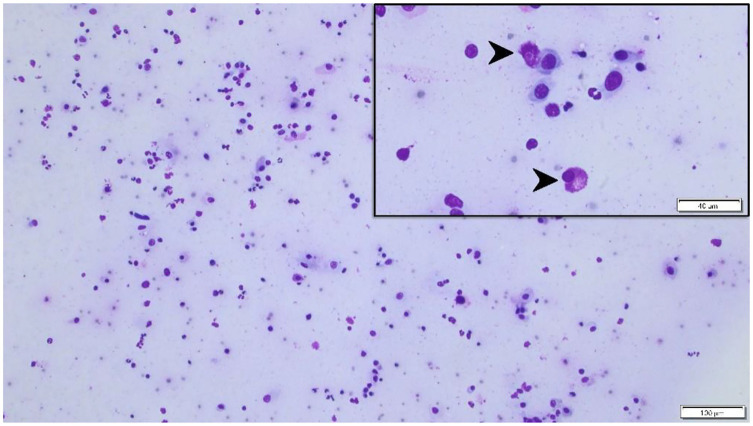
Cytological specimen of the left corneal surface (Wright Giemsa stain) highlighting a mixed, predominantly eosinophilic inflammation (black arrowheads) with few mast cells and lymphocytes. Bar = 40 μm (top right insert) or 100 μm (background image).

On presentation to a board-certified veterinary ophthalmologist (LS), physical examination and vital parameters were within normal limits. The cat appeared hesitant in the examination room (difficulty navigating, unable to track cotton balls) and the menace response was absent OU. Dazzle and palpebral reflexes were intact although pupillary light reflexes were not able to be assessed due to the severity of the corneal opacification OU. Mild blepharospasm and seromucoid to mucopurulent discharge were present OU. The conjunctiva had diffuse moderate hyperemia and mild chemosis OU. Dense fibrovascular ingrowth affected 100% of the anterior cornea OU, in addition to white raised plaques present on the corneal surface OU (masked by the third eyelid OD) (Figures 1A,B). Ocular diagnostic testing showed that intraocular pressures (TonoVet, Jorgensen Laboratories) were normal (23 mmHg OD, 24 mmHg OS), Schirmer tear test-1 (Merck Animal Health) results were normal (16 mm/min OD, 19 mm/min OS) (9, 10), and multifocal punctate areas of fluorescein uptake were noted OU. Cytological evaluation of the white corneal plaques revealed a mixed, predominately eosinophilic inflammation with few mast cells and lymphocytes (Figure 2). Clinical diagnoses were severe EK with punctate corneal ulcerations and moderate conjunctivitis OU. Treatment was initiated with triamcinolone acetonide (Vetalog, Boehringer Ingelheim Vetmedica, St. Joseph, MO) administered subcutaneously at 0.2 mg/kg, as well as 0.3% ciprofloxacin ophthalmic solution (Ciloxan, Alcon Laboratories, Fort Worth, TX; 1 drop OU q12h) and two topical compounded medications started 5 days later upon receipt: 0.5% tacrolimus aqueous solution (Stokes pharmacy, Mount Laurel, NJ; 1 drop OU q6h) and 0.5% cidofovir aqueous solution (Stokes Pharmacy, Mount Laurel, NJ; 1 drop OU q12h).

On Day 1 (i.e., 24 h following triamcinolone injection), telephonic update with the owner revealed that the cat's visual behavior and ocular comfort were greatly improved, as well as the tolerance to handling for topical drug administration. On Day 11, recheck examination showed marked improvement in vision (navigation in exam room, intact menace response OU) and ocular comfort level (no blepharospasm), with notable reduction in ocular discharge and conjunctivitis severity OU (mild residual hyperemia). Keratitis was greatly improved with resolution of corneal plaques and epithelial defects OU (no fluorescein uptake), as well as marked reduction in the fibrovascular ingrowth (mild residual corneal vascularization) allowing for clear visualization of the inside of the eye OU (Figures 1C,D). A subcutaneous injection of triamcinolone acetonide (0.2 mg/kg) was repeated, 0.3% ciprofloxacin was discontinued, 0.5% cidofovir was continued q12h for 2 weeks, and 0.5% tacrolimus was slowly tapered (q8h for 3 weeks then q12h until recheck).

Recheck evaluation on Day 38 revealed resolution of conjunctivitis and ocular discharge, and near resolution of the keratitis OU (Figures 1E,F). Only thin superficial vessels were present in the cornea OU, most of which were hypoperfused (“ghost” vessels). Intraocular pressures (20 mmHg OD, 25 mmHg OS) and Schirmer tear test-1 results (20 mm/min OD, 25 mm/min OS) were normal. At home treatment involved 0.5% tacrolimus at q12h for 1 week, then q24h for 4 weeks, then q48h with instructions to discontinue administration 1 week before the following recheck.

**Figure 3:**
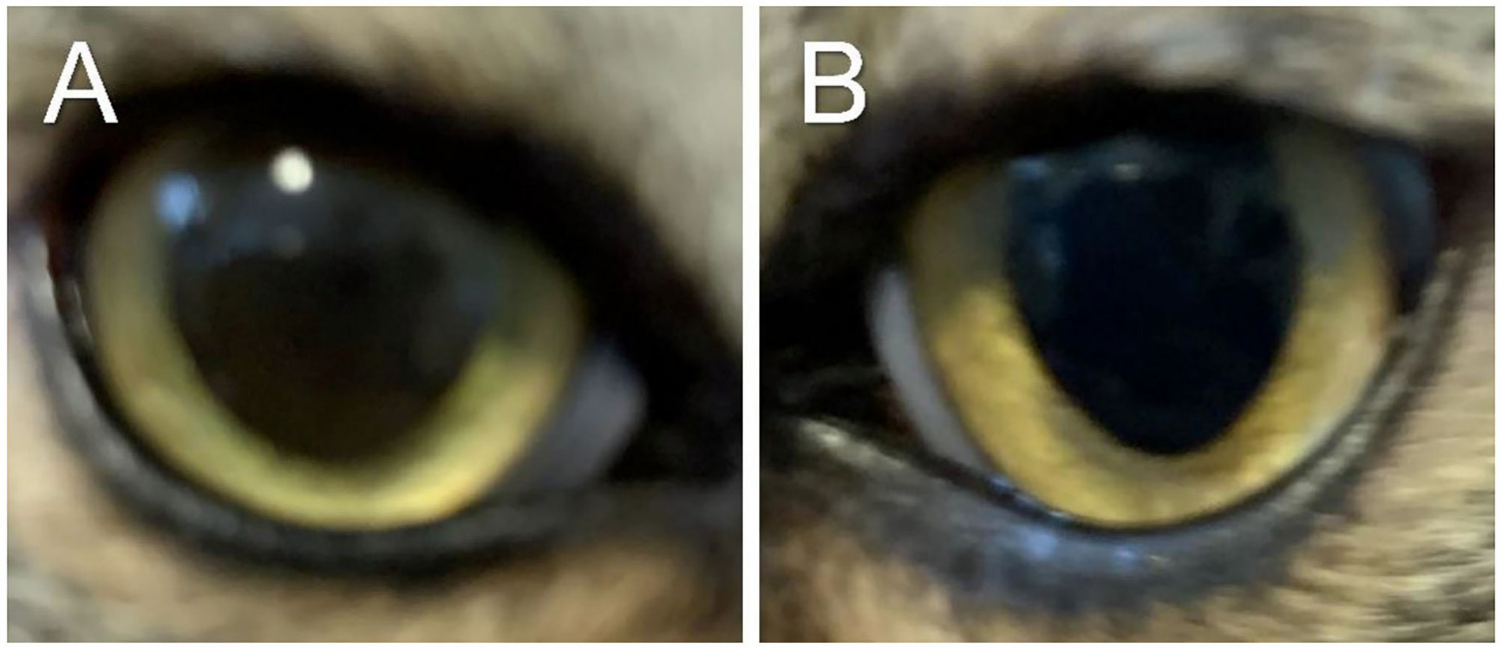
Clinical images of the right eye **(A)** and left eye **(B)** of a cat with historical eosinophilic keratitis in clinical remission. Images were taken by the owner using a smartphone on Day 374 following initial diagnosis, that is, 278 days after discontinuation of all therapies.

**Figure 4:**
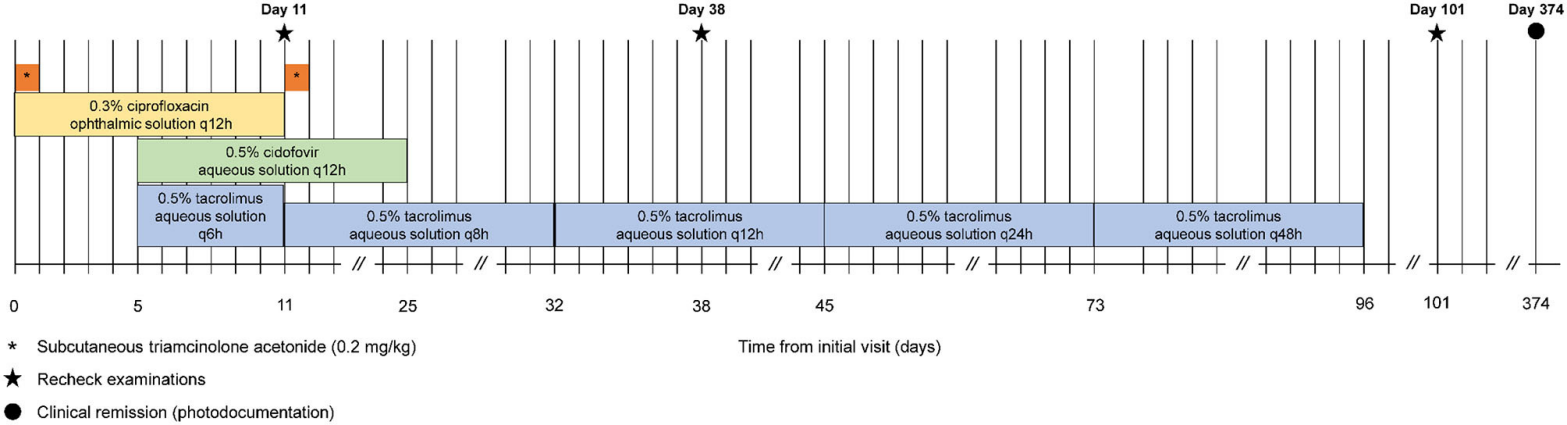
Timeline of the cat's clinical course and treatments.

The cat was re-examined on Day 101, off any medication for 5 days. The recheck visit confirmed resolution of ocular signs OU with only few hypoperfused superficial corneal vessels noticed on retro-illumination (Figures 1G,H). Throughout all visits, no systemic adverse effects (e.g., flare up of herpetic disease such as sneezing or nasal discharge) or ocular irritation from compounded tacrolimus were reported by the owner. Further, phone and email communications with the owner confirmed long-term clinical remission (i.e., no recurrence of ocular surface disease) despite discontinuation of all therapies. The latest follow-up at time of manuscript writing was Day 374 (Figures 3, 4).

## Discussion

The present case report describes the successful management of severe EK in both eyes of a cat, highlighting two important features: (i) Use of triamcinolone acetonide as the primary method of immunomodulation (rather than conventional topical immunotherapy) to improve therapeutic compliance; and (ii) Clinical remission with no recurrence of keratitis despite discontinuation of immunomodulation after merely 3.2 months of therapy.

Triamcinolone acetonide is an intermediate acting glucocorticoid with negligible mineralocorticoid effects, considered approximately 7 times as potent as methylprednisone (11). Parenteral administration of triamcinolone provides corticosteroid effects for 7–15 days, and a second dose can be administered following this time if signs persist (12). Here, triamcinolone acetonide was injected subcutaneously at two visits separated by 11 days, providing a rapid and marked improvement in clinical signs and ocular comfort. Our findings are consistent with a study of EK in horses, where systemic corticosteroid use was associated with a significantly shorter time for resolution of clinical signs (4). Our work is also complementary to the recent study by Lucyshyn et al., in which subcutaneous administration of triamcinolone was deemed safe and as efficacious as conventional topical immunomodulation in cats with eosinophilic keratoconjunctivitis (13). However, two main differences exist between the two reports: (i) First, clinical remission (noted in our patient) was not described in any cat reported by Lucyshyn et al., a finding that could be partly explained by the higher intensity of topical therapy in this case report (i.e., high concentration and frequency of tacrolimus); (ii) Second, triamcinolone was administered at time of diagnosis in our feline patient, whereas triamcinolone was preceded by a course of antiviral therapy with famciclovir or 0.5% cidofovir (median 17 days, range 0–63 days) in Lucyshyn's study. The tentative goal of initiating antiviral therapy prior to corticotherapy was to reduce the likelihood of herpetic flare-up and corneal ulceration, however the authors acknowledged that simultaneous administration of triamcinolone and antiviral drug may be preferred (as described herein) to reduce time to disease resolution, cost, and number of recheck visits.

There are several notable advantages to the unique protocol described in our study. First, triamcinolone acetonide provided rapid and pronounced improvement in corneal inflammation, allowing for corneal epithelial defects to heal without the risks associated with topical immunomodulation (i.e., potentiation of infection, delayed re-epithelialization). Second, medication compliance from the patient (and consequently owner) was greatly enhanced by reducing ocular discomfort before initiation of topical medications at high frequency; indeed, administration of multiple eyedrops 2–3 times daily (or more) can be particularly challenging in a cat that is painful and hiding from the owner. Compliance is particularly relevant for EK as most cases of recurrences were reportedly associated with poor mediation adherence (2). In the present case, the owner struggled medicating the cat prior to the first visit, then reported excellent compliance as early as Day 1 given the substantial improvement in the cat's ocular comfort and general demeanor. Third, corticosteroid levels on the ocular surface are likely optimized with triamcinolone acetonide as compared to topical corticosteroid use; the former provides a controlled-release formulation that achieves sustained concentrations over an extended period (zero-order kinetics), while the latter provides high concentrations at delivery but rapid decline due to efficient drug removal via the nasolacrimal drainage apparatus (first-order kinetics) (14, 15). Systemic corticosteroid administration achieved quantifiable tear film concentrations in dogs (16), and the same is likely true in cats. Further research is needed to characterize triamcinolone pharmacokinetics in cats, assessing healthy subjects but also cats with conjunctivitis given the likelihood for higher tear film concentrations in eyes with compromised blood-tear barrier (17, 18).

Similar to other reports of cats receiving triamcinolone acetonide for EK or other conditions (13, 19), our patient did not experience notable adverse effects from systemic corticotherapy. However, this observation should be verified in future prospective studies that include systemic workup and diagnostic testing. Potential adverse effects of systemic corticotherapy in cats include (but not limited to) recrudescence of herpetic disease (20), plasma volume expansion (21) that could promote congestive heart failure in predisposed cats (22), and rarely, iatrogenic hyperadrenocorticism (23).

Maintenance immunotherapy was achieved with topical 0.5% tacrolimus aqueous solution, following an initial frequency of four times daily that was slowly tapered off over 3 months. Of note, the prescribed concentration (0.5%) was much higher than the typical dose of tacrolimus reported in previous ophthalmic studies (0.02%) (24), with the aim to provide an aggressive immunosuppressive therapy from the onset of medical management (discussed below). Further, tacrolimus was preferred over previously described 1.5% cyclosporine (2) for two reasons: (i) Tacrolimus is more potent than cyclosporine for reducing corneal neovascularization in patients with severe keratitis, as reported in dogs with keratoconjunctivitis sicca (24); and (ii) aqueous-based compounded tacrolimus is subjectively better tolerated than oil-based compounded cyclosporine in cats (authors' personal experience), thus reducing the risk of stopping therapy due to local adverse effects such as marginal blepharitis (2).

Monotherapy is generally effective in managing EK in cats, as described for topical 1.5% cyclosporine (2) or 0.5% megestrol acetate (5), although this approach is limited by incomplete success rate (11.4 and 12% non-responsive, respectively), relatively slow clinical improvement (>2–3 weeks), disease recurrence (22.6 and 33%, respectively), and the need to maintain cats on immunomodulation long-term to lifelong (2, 5). In contrast, the use of combined immunosuppressive drugs in the present case (systemic triamcinolone and topical tacrolimus) achieved a rapid improvement of EK (<11 days) that resulted in clinical remission following discontinuation of all medications by 3.2 months. There is mounting evidence that early and aggressive immunomodulation improves long-term clinical outcomes and disease prognosis in humans with diverse immune-mediated diseases (e.g., rheumatoid arthritis, Crohn's disease). For instance, human patients with Crohn's disease were significantly more likely to achieve clinical remission when receiving combination immunosuppressive therapy (60%) rather than conventional therapy with incremental use of immunomodulation drugs (35.9%) (25). The same may be true in cats with EK given the immune-mediated pathogenesis of this ocular condition (2, 5), although the findings of the present case report should be verified in future prospective controlled studies assessing a larger population of cats.

The main limitation of the study was the lack of diagnostic testing for FHV-1. FHV-1 is detected in over 76% of corneal scrapings from cats with EK (6), and its presence can complicate medical management due to potential herpetic flare up with immunosuppressive drug. The patient had historical upper respiratory infections during kittenhood, thus 0.5% cidofovir was initiated empirically to reduce the risk for potential FHV-1 re-activation despite the lack of confirmatory testing. Cidofovir was well-tolerated and discontinued 2 weeks after re-epithelialization of corneal ulcers.

## Concluding Remarks

The case report highlights the use of combination immunosuppressive therapy to achieve rapid resolution of clinical signs and greater chance for full recovery (clinical remission). Subcutaneous administration of triamcinolone acetonide improved ocular comfort and therapeutic compliance—a key component for long-term success—and resulted in excellent disease control in a rapid and effective manner.

## Data Availability Statement

The raw data supporting the conclusions of this article will be made available by the authors, without undue reservation.

## Ethics Statement

Ethical review and approval was not required for the animal study because the study describes the clinical management of a patient for a routine ocular condition. Written informed consent was obtained from the owner for the participation of their animal in this study.

## Author Contributions

LS examined the patient and conceived the medical management used in the study. AR and LS wrote the manuscript. All authors contributed to the article and approved the submitted version.

## Conflict of Interest

The authors declare that the research was conducted in the absence of any commercial or financial relationships that could be construed as a potential conflict of interest.
